# Recommendations for addressing the translational gap between experimental and clinical research on amyloid diseases

**DOI:** 10.1186/s12967-022-03420-9

**Published:** 2022-05-13

**Authors:** Miriam Solomon, Vito Foderà, Annette Eva Langkilde, Perry Elliott, Fabrizio Tagliavini, Trevor Forsyth, Oxana Klementieva, Vittorio Bellotti

**Affiliations:** 1 Department of Philosophy, Temple University, Philadelphia, PA USA; 2 Department of Pharmacy, University of Copenhagen, Copenhagen, Denmark; 3 Department of Drug Design and Pharmacology, University of Copenhagen, Copenhagen, Denmark; 4 Institute of Cardiovascular Science, University College London (UCL), London, UK; 5 Fondazione IRCCS Istituto Neurologico “Carlo Besta” (FINCB), Milan, Italy; 6 Lund Institute for Advanced Neutron and X‐ray Science (LINXS), 223 70 Lund, Sweden; 7 University College London (UCL), London, UK; 8 Faculty of Medicine, Lund University, SE-221 84 Lund, Sweden; 9 Direzione Scientifica, Fondazione IRCCS Policlinico San Matteo, Pavia, Italy

**Keywords:** Amyloid diseases, Translational medicine, Alzheimer’s disease, Negative results, Complexity, Heterogeneity

## Abstract

This paper is a report of recommendations for addressing translational challenges in amyloid disease research. They were developed during and following an international online workshop organized by the LINXS Institute of Advanced Neutron and X-Ray Science in March 2021. Key suggestions include improving cross-cultural communication between basic science and clinical research, increasing the influence of scientific societies and journals (vis-à-vis funding agencies and pharmaceutical companies), improving the dissemination of negative results, and strengthening the ethos of science.

## Introduction

Amyloidoses are generally late-onset diseases in which proteins, after decades of normal service in the body, lose their native function and start to aggregate into polymeric forms that are toxic for cells and tissues. In 1997, the Nobel laureate Max Perutz [1] used the term ‘chameleon molecules’ to describe proteins that are capable of adapting their shape to different environments. This challenged the long-held view that a unique protein sequence corresponds to a unique three-dimensional configuration. This chameleon character can result in both benefit and harm to the organism. In the case of amyloid diseases, proteins that are normally thought of as forming a specific native conformation aggregate into strong fibrils that interfere with normal function. Both basic and clinical research are challenged by the heterogeneity, complexity, and time course of amyloidogenesis. Like examples from oncology, rheumatology, and psychiatry (among other specialties), amyloidoses are what philosophers of science call “SCOTCH” (Significant Change Over Time, Complexity, and Heterogeneity) diseases.

The ways in which basic science has influenced medical progress in amyloidoses was extensively reviewed by Joel Buxbaum and Rheinold Linke in 2012 [2]. However, there has since been extraordinary progress in both basic science and clinical approaches to the diseases. Treatment of the disease is now possible for certain types of transthyretin amyloidosis. The first drug specifically targeting the molecular mechanism of the disease, through a stabilization of native state of the protein [3]**,** is now available and licensed for therapy in many countries. Although not curative in all patients it represents very important progress in modifying the natural history of a disease considered almost incurable until a few years ago. A second very promising and innovative approach uses the silencing of the expression of pathogenic protein through the deployment of oligonucleotide technology [4] or, as recently reported for the first time directly in patients, through the use of CRISPR-CAS9 gene silencing technology [5]. Knocking out the expression of the pathogenic protein has been made possible by the extraordinary progress in our capacity to safely manipulate genes in vivo over the last twenty years. These innovations were possible both because of fundamental research that allowed the identification of the pathogenic molecules and because of advances with in vivo genetic engineering (which were for some time a translational challenge). Pedro Costa identified transthyretin (TTR) as the constituent of amyloid in Portuguese familial polyneuropathy in 1978 [6]. Following this, it took 40 years for Costa’s pioneering work to be crowned by the development of treatments for ATTR amyloidosis which were unimaginable at the time.

There have of course been clinical trials of proposed drugs for other kinds of amyloidosis that have ended in negative findings. This is particularly so for drugs aimed at treating Alzheimer’s Disease. Even the recently FDA approved drug Aducanumab is likely to be clinically ineffective, and not all healthcare systems in the United States have decided to add the drug in their formulary [7]. Such disappointing results probably result from insufficient understanding of the complexity of disease mechanisms, and the related use of simplified in vitro and animal models that do not translate into clinical effectiveness in humans.

The “translational medicine” initiative started in the early 2000s (see, for example, the 2004 NIH Roadmap for Medical Research (https://grants.nih.gov/grants/guide/notice-files/NOT-RM-04-010.html)) recognizes the difficulties in moving from basic science to clinical intervention and seeks to accelerate the “bench to bedside” process through multidisciplinary collaborations that include both basic scientists and clinical researchers. A crucial aspect of this initiative is to overcome institutional obstacles to such collaborations (see [8], Chapter 7 for an account of the early history of translational medicine).

In March 2021 an online workshop (organized by the LINXS Institute of Advanced Neutron and X-Ray Science in Sweden) was convened through Zoom to discuss translational challenges specific to the amyloidosis research community. There were talks on the history of amyloidosis research and on the current state of amyloidosis research in the areas of cardiac amyloidosis, Alzheimer’s disease, leukoencephalopathy, biophysics, structural biology, molecular biology, and in vivo models. The discussion focused on the existing gap between experimental research and clinical practice and the progress needed in order to narrow (and/or prevent the expansion of) this gap. Clinicians and basic scientists gave short talks followed by discussions for four hours; also present was a philosopher of science/medicine with expertise in translational medicine.

The workshop was followed up with a multiple-choice questionnaire with five questions designed to gather participants’ opinions on the core issues, following the discussions at the meeting. This paper summarizes the major issues raised both during the conference and subsequently by the questionnaire, and makes recommendations for addressing the translational gap. Thus, this paper represents the results of deliberations of well-informed participants. While it is not a “consensus document” in the sense that it presents a single statement on which all agree, it should help focus further discussion on the concerns that were thought to be most important.

**Fig. 1:**
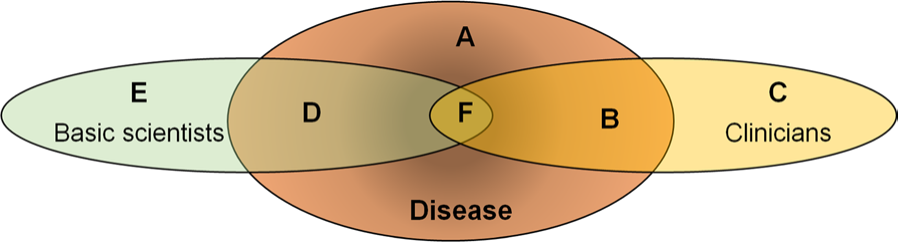
Schematic representation of the expertise workshop participates. **A** The coloured area **A** represents amyloid diseases. **B** Disease relevant expertise of clinical scientists. **C** Clinical expertise in other areas **D** Basic scientists whose major research interest is in amyloid disease **E** Basic scientists in other research areas **F** Overlap of clinical and basic research expertise in the area of amyloid diseases

From discussion and presentations at least 5 major categories of professionals were identified. They are schematically shown in Fig. 1. Specifically, the area **A** represents those working on amyloid diseases. Area **B** represents the clinicians working at the front line with amyloid disease. Several have long-term experience with very specific aspects of the disease and many are working in specialized centres developing best practices in diagnosis and treatment. Some clinicians are also directly involved in carrying out preclinical studies (area **F**). This is a relatively small category since clinicians with the necessary training in basic science are now quite rare. Area **C** represents clinicians in different disciplines focusing mostly on the function of single organ, such as cardiologists, who care for patients with localised manifestation of amyloid diseases. They do not deal with the complexity of systemic diseases which result in multi-organ involvement. **D** represents basic scientists whose major research interest is in the mechanisms of amyloid formation. This includes a growing number of chemists, biochemists, biologists and biophysicists—very few of whom are medically qualified. These scientists are particularly involved in creating preclinical models of amyloid diseases in vitro and in vivo and in studying molecular structures and functions using state-of-the-art techniques such as X-ray and neutron diffraction, cryo-electron microscopy, NMR spectroscopy, mass spectrometry, molecular simulations, AI protein structure prediction, etc. A small number of these scientists overlap into **F,** alongside the few clinical researchers with sufficient knowledge of fundamental science. This is a particularly important overlap that allows a direct interaction with clinicians on diagnosis and pre-clinical studies, including drug development.

An increasing number of non-clinical (basic, fundamental) scientists may have limited interest in amyloid-related disease itself and focus on related in vitro models, perhaps with methodological, technical, or computational bents. Despite being partially abstracted from the clinical context, this group nevertheless works on crucial and fundamental questions of high significance to the dynamics of protein aggregation and the structure of amyloid materials (space **E**)**.**

Figure 1 is, of course, an oversimplification, but it emphasises the wide range of expertise amongst those working on amyloid diseases. The perspectives of communities **B** through **F** were reflected at the workshop and within the survey. The area in which translational issues are paramount is area **F**. One the major concerns that ran through the entire discussion was the conflict between the increasingly interdisciplinary needs of training for translational approaches at a time when expertise is becoming increasingly specialised.

### The multiple-choice questionnaire: questions and results

After the end of the meeting a multiple-choice questionnaire with seven questions was circulated to participants to gather different opinions. The participants were roughly equally distributed between basic scientists working in area D and physicians working in area B. 23 out of 64 participants returned the questionnaire, and the results are reported, and commented on, in the pie charts shown below. Although the numbers and methods, in this case, are not analyzed for statistical significance, the results are reported as a qualitative exploration of the more major challenges for translational research on amyloid diseases.

**Fig. 2:**
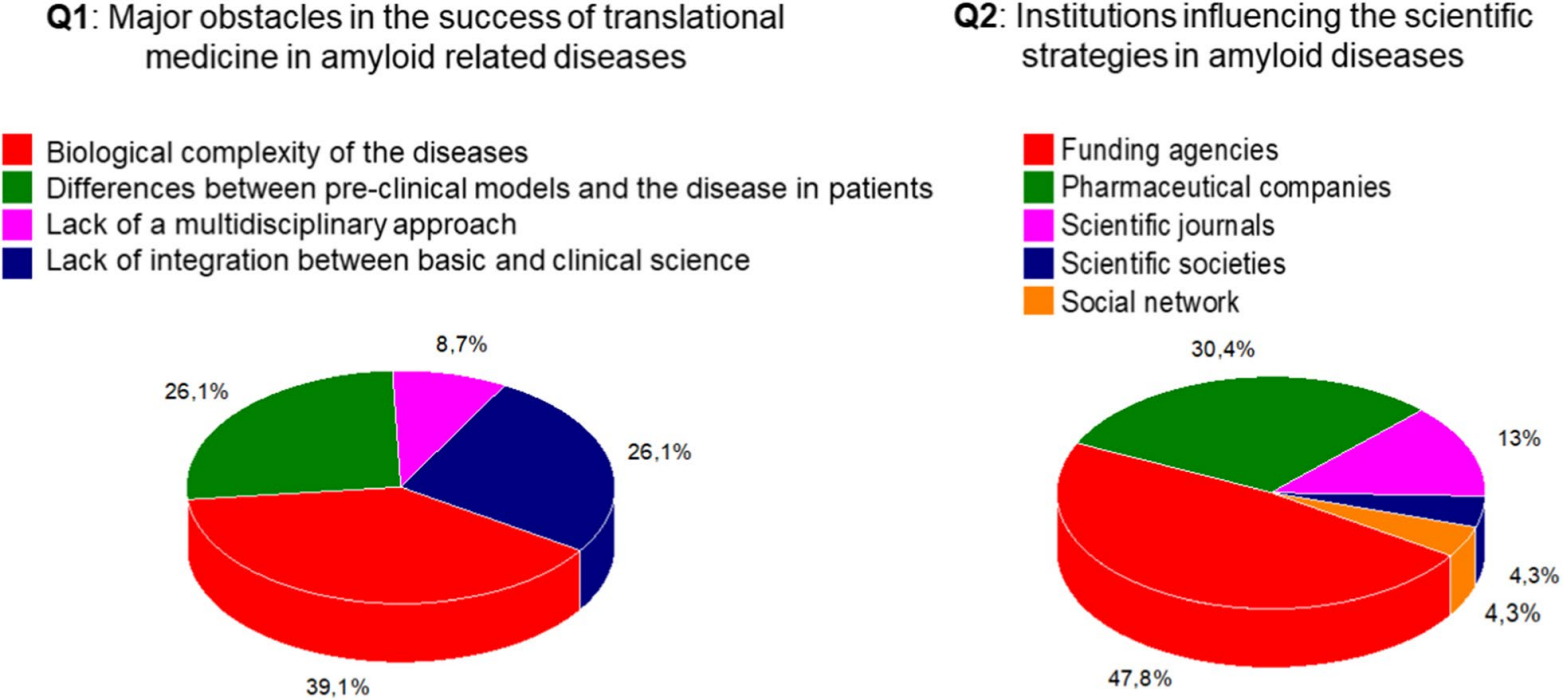
Major obstacles and influence of scientific strategies in amyloid diseases

**First question** (Fig. 2 Q1): Which is the major obstacle to the success of translational medicine in amyloid related diseases? The following four answers were proposed: Biological complexity of the diseases/Lack of integration between basic and clinical science/Biological differences between pre-clinical models and the disease in patients/Lack of multidisciplinary approaches.) A significant proportion of the participants (almost 40%) reported that difficulties in translation are intrinsically related to the biological complexity of the disease. The research findings of multifactorial pathogenic mechanisms of the disease are evidence for this complexity. A majority of respondents (red plus yellow areas making 52.2%) attributed the difficulties to differences between approaches within basic and clinical sciences—an issue that is aggravated by complexity. Only 8.7% responded that the difficulty was the lack of a multidisciplinary approach, suggesting that disciplinary differences are not the crucial issue.

**Second question** (Fig. 2 Q2): List in order of major impact the institutions influencing the scientific strategies in amyloid diseases. Pick the two most important. (Proposed answers: Scientific Journals/Media/Social Network/Scientific societies/Pharmaceutical companies/Patients associations/Funding agencies).

According to the participants the two major stakeholders influencing the scientific strategies are funding agencies (43.5%) and pharmaceutical companies (30.4%). Scientific journals have a lower level of influence (13%). Scientific societies, media, and patients’ associations are perceived to have a low level of influence.

**Fig. 3:**
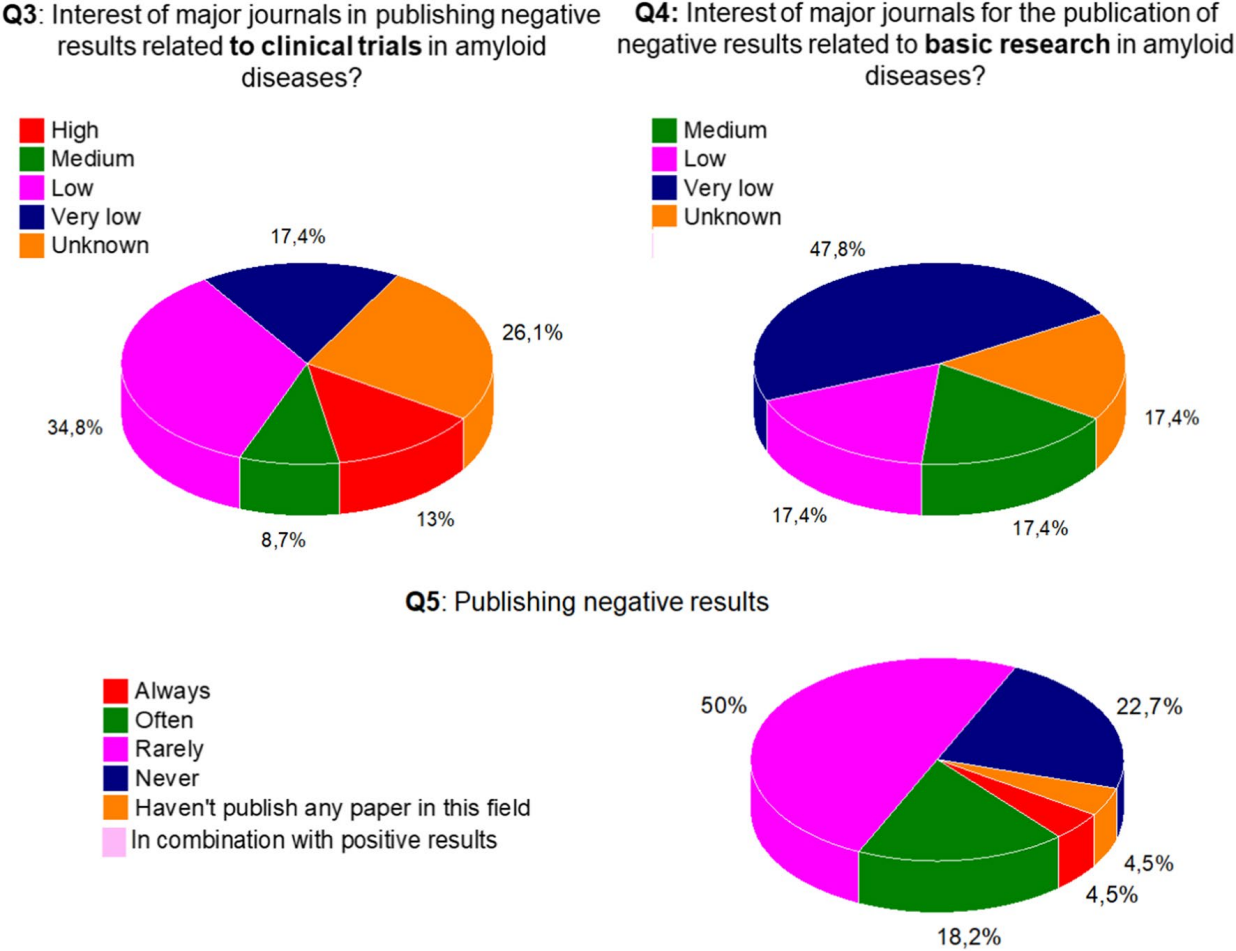
Publishing negative results

Using **third and fourth questions** (Fig. 3): the perceived interest of major journals in publishing negative results related to clinical trials was compared with the perceived interest of major journals in publishing negative results related to basic research in amyloid diseases (Fig. 3). Proposed answers: High, Medium, Low, Very Low, Unknown). The third and fourth questions ask for an opinion about the interest of journals in publishing negative results in the amyloid field, for clinical trials and for basic research. Both report low interest in publishing negative results, but the perceived interest in publishing negative results in basic research is *even lower* than for clinical trials.

**Fifth question**: Do you publish negative results? Almost 70% of particpants answer this question as “never or rarely,” suggesting that a huge amount of work produced in our laboratories or clinical centers remains inaccessible. That 17.4% say that they often publish negative results is intriguing and worth further exploration if more publication of negative results is recommended.

**Fig. 4:**
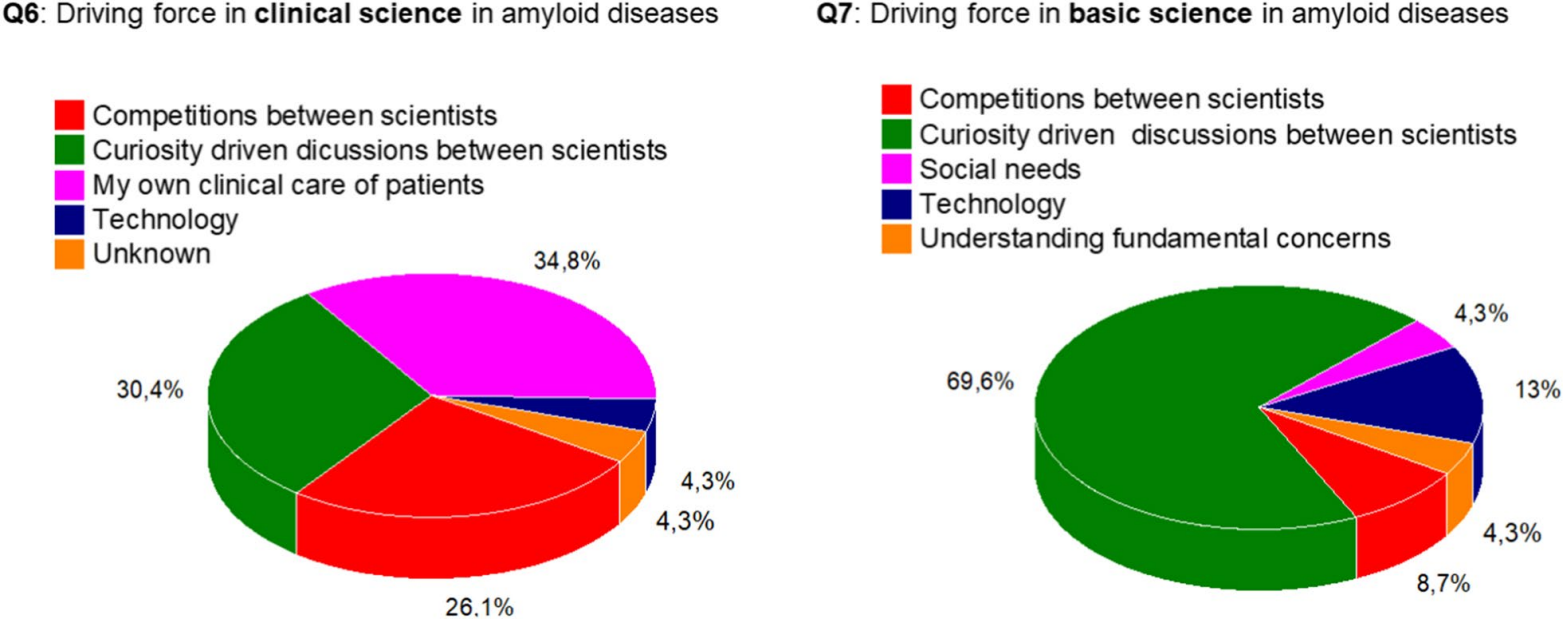
Driving force in clinical and basic science in amyloid diseases

**Sixth and seventh questions:** The major driving forces in clinical science were compared with those of basic science of amyloid diseases (Fig. 4). The major driving force in basic science is perceived to be curiosity driven questions (69.6%) whereas in clinical science the driving forces are social needs (39.1%), competition between scientists (26.1%) and curiosity driven questions (21.7%).

## Discussion

The first question addresses the translational challenges in amyloid related diseases and explores the underlying reasons. The responses echoed the workshop discussion: amyloid diseases (like, for example, cancer and schizophrenia) involve highly complex and variable underlying mechanisms. Laboratory models tend to make simplifying assumptions, and these assumptions often fail when laboratory results are extended to the clinical context. The risk of oversimplification scales with the in vivo complexity of the mechanisms.

In addition, the challenge of translating basic scientific results to clinical applications requires the formation of effective bridges between two rather disparate domains that have somewhat different epistemic cultures. It is rare to find someone with skills in both (although some with MD-PhD training do fall in area **F** of Fig. 1), and collaboration between individuals with different background training can be challenging. It is important to learn how to tackle this on a broad scale, since new insights about amyloid disease may come from other areas in basic science (area **E**) or other areas in clinical medicine (area **C**): *e.g.* techniques from CRISPR-CAS, insights from the study of biomechanical forces in protein amyloidogenesis from Von Willebrand disease, AI in protein structure prediction.

The second question asks about the institutional factors that exacerbate the scientific problems of translation. The perception of the respondents is that the priorities of funding agencies and pharmaceutical companies have a crucial role in influencing research directions and may even distort fundamental scientific strategy. This is consistent with the fact that the high cost of basic and clinical research commits scientists to the constant and demanding preparation of grant proposals and the fostering of industry collaborations. At present, both funding agencies and pharmaceutical companies pressure scientists towards translational work. While in some cases this may generate excellent results, in other cases it can be premature or even contrived. When knowledge of disease mechanisms is incomplete, it may not be reasonable to expect translational success and it may be more productive in the long term to invest in basic research that does not have immediate translational impact. Funding agencies and the pharmaceutical sector are not always driven by the level of scientific information that might be hoped for and may even discourage essential basic research needed as an essential precursor to effective clinical practice.

It should also be pointed out that funding agencies and pharmaceutical companies are *not* completely independent assessors of scientific research. It is not unknown for “opinion leaders” to act both as reviewers of public grants and also as board members for pharmaceutical companies. In practice the policies and politics of funding agencies and pharmaceutical companies are entangled. The perception that short-term academic and pharmaceutical funding has a much stronger impact on the prioritisation of research than do scientific societies and the scientific literature is highly problematic and undermines confidence in collective rational action by the scientific and clinical communities. If scientific journals and learned societies in amyloidosis were revitalized and better established, this situation could be changed. In connection with this, it is also worth noting that the media is seen to have low impact. This may, at least in part, be a consequence of the weak role that scientific societies and patient associations play in creating social awareness of amyloid diseases.

There is a clear perception that patient associations are less influential in elevating the profile of amyloidosis than they do for other diseases. For example, in the case of cystic fibrosis a major role is played by highly motivated parents of sick children not only in supporting research but also in influencing research directions. Such patient advocacy is very much weaker for amyloid diseases, presumably because they are mostly diseases of the elderly. Improved advocacy on the part of patients, families, and researchers, as well as addressing prejudices about aging could improve this situation.

The third, fourth, and fifth questions address the publication of negative results. While this is of much broader scientific concern, the high rate of translational failure makes the handling of negative results especially important to this discussion.

It is striking to note that the perceived interest in publishing negative results in basic research is even lower than it is for clinical trials. This difference may be due to recent initiatives to preregister clinical trials and report their results (see for example https://clinicaltrials.gov/). There have been no similar initiatives (*e.g.* to register hypotheses under test) in basic research, perhaps because the impetus to preregister clinical trials and report their results has more to do with concerns about pharmaceutical company bias than it has with the desire to produce a public record of negative results. However, it is as important, scientifically, to publicise negative results in basic research as it is in clinical research since it may improve efficacy and direct energy and resources to more promising strategies. In addition, such efficacy increases responsible consumption of resources within the UN Sustainable Development goals.

A further issue, not explored in the questionnaire, is that negative results, when they are published, may end up being reported in lower impact journals. If so, the low number of citations of these journals suggests that they are being less read. The ethos associated with funding, and the metrics now almost universally used in assessing academic impact and professional progress, inevitably propels scientists to read and publish in high impact journals. It would seem that the large quantity of journal output in the area (2.5 million in 2018) is heavily polarized in this way, leaving negative results omitted or obscured. It is of course difficult to know how best to address this problem but various options exist including the formation of a repository of negative results or even the extension of supplementary information sections of published articles. More than ever, the technology to make this type of information readily accessible and indexable is readily available, but developing it has not been adequately prioritized.

## Recommendations

During the preparations for the workshop, Vittorio Belotti described the experience of doing research in amyloid diseases as “like being in a car that we don’t know how to drive.” This phrase captures the sense that there are larger institutional, political, and financial forces driving the science, and that scientists themselves feel somewhat powerless. These recommendations are about what can be done to take back that power.

- Improved cross-cultural communication between basic science and clinical medicine would help address translational challenges. This communication could be in the form of more “bilingual” people in area F (more MD PhDs perhaps) or could be the result of better communication between people with different training and skills. In general, greater communication between people in different fields would help avoid the pitfalls of high specialization. The same sort of mentality needs increasingly to be built into teaching curricula.
- Scientists working with models (whether model organisms or model in vitro systems) should keep in mind the differences between models and the human disease whose understanding is the goal of inquiry.
- Research priorities/strategies should come from a variety of sources, not only funding agencies and pharmaceutical companies. One possibility is to strengthen the role of scientific societies, scientific journals, and patient associations and empower them to influence the direction of research. Scientific societies and scientific journals represent the scientific community more than funding agencies and pharmaceutical companies do at this time. Patient associations can also play important roles and sometimes harness media attention.
- Negative results in both basic research and clinical trials need better dissemination. The present system in which positive results receive all the rewards is harmful to both science and scientists (the Matthew effect [9]). If negative results were treated as appropriately important, scientists might risk more ambitious theories.
- Excessive emphasis on scientometric parameters such as impact factors and H factors is harmful to science and scientists. The growing tendency to read abstracts instead of whole papers also impedes dissemination of ideas. The impact of choice of metrics is of course not only on translational research but also more broadly on recruitment, funding, assessment, etc. Detailed consideration is beyond the scope of this paper.
- Competition can energize scientists, but it can also encourage secrecy, which damages collaboration and may be detrimental to the progress of science. Concern was expressed (at the conference) about competition between countries, and even competition between funding agencies.
- The traditional ethos of science which includes regarding science as a communal and cooperative project, critical interaction between scientists, the importance of lack of financial bias, and striving for scientific objectivity (an early account is [10]) is difficult to sustain in present day circumstances. Is it time for an updated ethos, perhaps one that would draw on more recent normative work that incorporates processes intended to address bias? (e.g. [11])

## Data Availability

The datasets used during the current study are available from the corresponding author on reasonable request.

## References

[1] Perutz MF (1997). Mutations make enzyme polymerize. Nature.

[2] Buxbaum JN, Linke RP (2012). A molecular history of the amyloidoses. J Mol Biol.

[3] David A, Hawkins PN, Polydefkis M (2018). Oligonucleotide drugs for transthyretin amyloidosis. N Engl J Med.

[4] Rapezzi C, Elliott P, Damy T, Nativi-Nicolau J, Berk JL, Velazquez EJ (2021). Efficacy of tafamidis in patients with hereditary and wild-type transthyretin amyloid cardiomyopathy. JACC: Heart Failure.

[5] Gillmore JD, Gane E, Taubel J, Kao J, Fontana M, Maitland ML (2021). CRISPR-Cas9 In Vivo gene editing for transthyretin amyloidosis. N Engl J Med.

[6] Costa PP, Figueira AS, Bravo FR (1978). Amyloid fibril protein related to prealbumin in familial amyloidotic polyneuropathy. Proc Natl Acad Sci.

[7] Tampi RR, Forester BP, Agronin M (2021). Aducanumab: evidence from clinical trial data and controversies. Drugs In Context.

[8] Solomon M (2015). Making medical knowledge.

[9] Merton RK (1968). The Matthew effect in science. The reward and communication systems of science are considered. Science.

[10] Merton RK. The sociology of science: theoretical and empirical incvestigations. 4. Dr. Chicago: Univ. of Chicago Pr; 1974. ISBN: 9780226520926

[11] Longino HE. Science as social knowledge: values and objectivity in scientific inquiry. Princeton, N.J: Princeton University Press; 1990. 10.2307/j.ctvx5wbfz.

